# Impact fact(or) fiction?

**DOI:** 10.1093/braincomms/fcac261

**Published:** 2022-11-08

**Authors:** Tara L Spires-Jones, David Belin

**Affiliations:** Edinburgh, UK; Cambridge, UK

Welcome to Volume 4 Issue 6 of *Brain Communications*. We launched the journal in April 2019, meaning we are 3.5 years old now and halfway through the 7-year tenure of the first editorial team. A question we are often asked by authors is ‘What is your impact factor?’. In one way, this is a very simple question to answer—we do not have one yet. In another way, this question opens a can of worms that all scientists, perhaps even more particularly those in the early stages of their careers, struggle with at some level.

Impact factor is a metric of journal citations calculated by the for-profit company Clarivate Analytics. Impact factor is a ratio between citations received and the number of citable papers published in a journal. For example, Clarivate’s website states that the 2021 impact factor of a journal was calculated as the cites in 2020 to items published in 2018 and 2019 divided by the total number of citable items published in 2018 and 2019.^1^ As you will know, impact factor is used as a measurement of how important a journal is, based on the logic that high average citations per paper means ‘good’ papers are published in the journal. The impact factor was developed long before the invention of the World Wide Web, at a time when librarians had to decide which journals to subscribe to and researchers had to browse manually through tables of contents of each issue of each journal in institution libraries. While there is nothing intrinsically wrong with knowing the average citations per paper in a journal, there is considerable controversy over the impact of impact factor (pun intended).

One problem with using impact factor as a metric is that people too often generalize the average citations to assess the quality of individual papers. Many of us have experienced direct pressure to publish in ‘high impact’, or top tier, journals, which are often the property of for-profit companies, even though having a paper in a high impact factor journal does not guarantee that it will be either highly cited or that it will be of high quality. Indeed, an analysis posted on bioRxiv by Larivière *et al.*^2^ shows that the citation of individual papers cannot be inferred from journal impact factor. Instead, the majority of papers in a given journal receive fewer citations than the average citation the journal attracts which is skewed, driven by some very highly cited outliers. When calculating the ratio between the citations a paper attracts and the product of the impact factor and the number of years since publication, it is easy to establish whether a particular paper contributes to the impact factor of the journal or if instead, it drags it down.

In spite of these clear confounds, whether intentionally or unintentionally, researchers on panels evaluating grant applications, hiring, or promotions have long evaluated the scholarly achievements of their peers in their field by the impact factor of the journals where their research was published. To begin to counteract this bias, many prominent scientists, research funders, and journal editors have signed up to the San Francisco Declaration on Research Assessment which aims to stop impact factor being used to assess individual scientists in favour of requiring that each paper be judged on its own merits.^3^

Another problem associated with chasing high impact factor journals, in our view, is that this practice promotes tunnel vision of what impactful contemporary research is. Focusing on high-impact journals as a driver of the ‘impact’ of research concentrates power in the hands of a few ‘gate-keeper’ editors and reviewers, while discouraging replication and robust, rigorous incremental science, which are fundamental to advancing knowledge using the scientific process. Most journals with high impact factors only want to publish very ‘novel’ (the definition of which is highly debatable), flashy work that will be highly cited. Since most ‘credit’ and citations tend to favour the first paper to publish a new idea, replicating results in different populations or model systems are often difficult to publish in these high impact factor journals. One of our core goals at *Brain Communications* is to facilitate a high standard of rigour in our published papers, and we welcome incremental work, negative results or replication studies, as long as the research is robustly conducted.

Thus, we are not driven at all to aim for a high impact factor, and we are thrilled that people are choosing to publish in our journal and that so many are willing to offer expertise in our transparent and constructive peer review process. It is also a real pleasure and a source of pride for us when we see our papers are cited, as it is an indicator that people are reading the valuable work published in our e-pages.

We will likely be bestowed an impact factor next year, which is great from the perspective that we will have an answer for authors to this question. But, we will be acutely aware that while impact factor is one informative metric to have for our journal, it is not important to our goals, and it has for too long generated undue stress and pressure in the scientific community and given privately owned journals too much influence over the careers and research of scientists, by handling the lottery of who gets into the big impact factor journals.

The cover for this issue comes from Feltrin *et al.*^4^ and shows a precision medicine targeting approach for transcranial magnetic resonance–guided high intensity focused ultrasound.
